# Mediators implementation and delivery: the falls management exercise programme (FaME)

**DOI:** 10.1186/s12913-025-13550-7

**Published:** 2025-10-22

**Authors:** Fay Manning, Jodi P. Ventre, Grace Brough, Helen Hawley-Hague, Claire Hulme, Denise Kendrick, Pip Logan, Aseel Mahmoud, Tahir Masud, Elizabeth Orton, Dawn A. Skelton, Stephen Timmons, Chris Todd, Victoria A. Goodwin

**Affiliations:** 1 National Institute for Health and Care Research Peninsula Applied Research Collaboration, University of Exeter Medical School, Exeter, EX1 2LU UK; 2 Department of Health Care Professionals, Faculty of Health and Life Sciences, Exeter, EX1 2LU UK; 3 NIHR ARC-Greater Manchester, School of Health Sciences, Faculty of Biology, Medicine and Health, The University of Manchester, Manchester, UK; 4 School of Medicine, University of Nottingham, Nottingham, UK; 5 University of Queensland, Brisbane, Australia; 6 Nottingham University Hospital NHS Trust, Nottingham, UK; 7 Research Centre for Health (ReaCH), School of Health and Life Sciences, Glasgow Caledonian University, Glasgow, UK; 8 Business School, University of Nottingham, Nottingham, UK; 9 Manchester Academic Health Science Centre, Manchester, UK; 10 Manchester University NHS Foundation Trust, Manchester, UK

**Keywords:** Falls, Exercise, Implementation, Fidelity, Mixed methods, Older adults

## Abstract

**Background:**

When implementing multicomponent interventions, fidelity to the intervention is key for reproducible outcomes. This study investigates the main influences on the fidelity (implementation strategy fidelity and intervention delivery fidelity) of the community-based Falls Management Exercise (FaME) programmes in three different areas of England.

**Methods:**

FaME classes across Greater Manchester (GM), Devon and the East Midlands (EM) were studied between 2021 and 23 using a mixed-methodological approach. Data sources included interviews, observations of FaME classes, field notes, and communities of practice recordings.

**Results:**

Forty interviews were conducted with stakeholders, providers and class attendees. Additionally, twenty-one class observations were conducted. Triangulated quantitative and qualitative data revealed issues with fidelity to the FaME programme, especially in sites with limited oversight roles/structures and limited funding. There was a lack of understanding and clarity of essential components which impacted both implementation strategy and intervention delivery fidelity. The conceptual map developed highlights the importance of mediators of fidelity in relation to implementation and delivery, including: economic influence, organisational influence, oversight roles, fidelity evaluation, participant responsiveness, essential components and knowledge, training and professionalism.

**Conclusion:**

Despite a recognised need for implementing evidence-based falls prevention programmes, a lack of sufficient funding, formalised oversight roles/structures and understanding of essential intervention components is associated with lower fidelity to the intervention. Unchecked by local monitoring, this can lead to an incremental migration of delivery away from the evidence base. We recommend: (i) providers have clarity on essential intervention components, (ii) standardised fidelity monitoring based on essential components, and (iii) effective local oversight roles and structures.

**Supplementary Information:**

The online version contains supplementary material available at 10.1186/s12913-025-13550-7.

**Table:** 

Text box 1. Contributions to the literature
• This study highlights key mediators that influence both implementation strategy and intervention delivery fidelity of complex interventions in community settings.
• The large mixed-methods study identifies the impact of organisational factors, oversight structures, and knowledge gaps on the fidelity of evidence-based falls prevention programmes.
• The findings emphasise the risks of intervention delivery drift in the absence of structured fidelity evaluation and oversight mechanisms.
• By identifying the need for greater clarity around essential components, the use of standardised fidelity assessments, and built-in oversight mechanisms, this study provides intervention-specific guidance to support improved implementation of FaME programmes in real-world public health settings.

## Background

Due to an ageing population, falls in older adults are an increasing public health issue, impacting the individual (negative effects on function, independence and quality of life), and society (health related costs and care burden), highlighting the need for prevention [1]. Strength and balance exercise programmes are recommended by the World Falls Guidelines and the National Institute for Health and Care Excellence (NICE) for falls prevention for older people living in the community with a low to moderate risk of falls [1, 2]. These recommendations are based on a broad evidence-base including a 2019 Cochrane systematic review which reported that community-based exercises aimed at improving balance and functional strength reduce the rate of falls by 24% [3]. 

FaME is a 24-week progressive exercise programme involving weekly group exercise led by Postural Stability Instructors (PSIs) and unsupervised home-based exercises to ensure an effective dose (50 h +, at least 2 h per week). It is a multi-component exercise programme which includes strength, endurance, flexibility and highly challenging dynamic training, alongside adapted Tai Chi and getting down to and up from the floor [4]. In a randomised controlled trial, the Falls Management Exercise (FaME) programme increased physical activity, improved balance confidence, and reduced the rate of falls amongst community-dwelling older adults by 31% [5]. When translated into routine practice, FaME remains effective but with more modest outcomes (24.5% reduced rate of falls at programme end, 23.8% at 6-month follow-up) [6]. 

The degree to which programmes are implemented as intended by the programme developers [7], is an important measure to understand how well evidence-based interventions are translated into practice (process of converting scientific evidence into clinical settings). In this paper, we distinguish between two related but distinct forms of fidelity. First, ‘implementation strategy fidelity’ refers to the extent to which the activities undertaken to support and embed the intervention, such as staff training, organisational changes and resource allocation, were enacted as intended. The purpose of these strategies is to ensure the intervention is integrated into practice in a way that enables it to achieve the outcomes demonstrated in prior trials. While fidelity to strategies is important, they may also be adapted to address local needs and contexts, which can influence both how strategies are implemented and their effectiveness [8]. Second, ‘intervention delivery fidelity’ concerns the extent to which the intervention itself was delivered to participants as intended, including adherence to session content structure and delivery quality.

A reduction in either fidelity, for example, may explain why proven interventions appear to be less effective in practice. That said, there are multiple reasons why an evidence-based intervention which has been shown to be effective in clinical trials may need to be adapted for delivery in real-world settings.

The Conceptual Framework for Implementation Fidelity (CFIF) outlines four moderators which influence or moderate the degree to which an intervention is implemented with fidelity [9]. These moderators include intervention complexity, facilitation strategies, quality of delivery and participant responsiveness, as complex inter-related factors. Lara et al., [10] discussed these moderators further, describing what they refer to as the ‘black cloud of translation’, illustrating the complex interrelationship and contextual influences, expanding the themes of Carroll et al.’s framework to include ‘real-life’ elements such as culture, politics and infrastructure.

A previous mixed-methods study, the PhISICAL study [11], described the fidelity of 29 FaME programmes in the East Midlands region of England. Instructor interviews, observational data and participant outcomes reported themes relating to the four moderators described in the CFIF, however, these primarily related to the in-class delivery of the FaME programme with minimal insights into wider geographical and local contextual factors [9, 11]. 

As part of a wider programme of work (FLEXI- FaLls EXercise Implementation [12]), this study aimed to investigate the main influences on the fidelity (implementation strategy fidelity and intervention delivery fidelity) of FaME, the community-based falls prevention intervention in different geographical areas of England.

A mixed-methods design was chosen to enable both a broad examination of fidelity across sites through quantitative observations and a more in-depth exploration of the contextual and implementation factors through qualitative methods. This approach was essential to capture the complexity of real-world delivery and to allow triangulation between different data sources to understand how and why fidelity varied across settings.

## Methods

A parallel qualitative-quantitative mixed-methods design and convergent data analysis was utilised [13] to address the following objectives (i) to assess the fidelity of FaME delivery using a structured checklist, and (ii) to understand influences on both implementation strategy and delivery fidelity, across three regions of England between 2021 and 23. The three regions (Devon in the South West, Greater Manchester [GM] in the North West and Leicestershire and Derby City in the East Midlands [EM]) were chosen for their diverse population demographics, socioeconomic landscapes and provision of the FaME intervention including both existing (running pre-study), and new provision (set up during the study). This allowed for exploration of different stages of the intervention, as well as revising a previously studied site (EM).

MMR-RHS reporting guidelines have been utilised due to the mixed-methods [see Additional file 1].

**Fig. 1:**
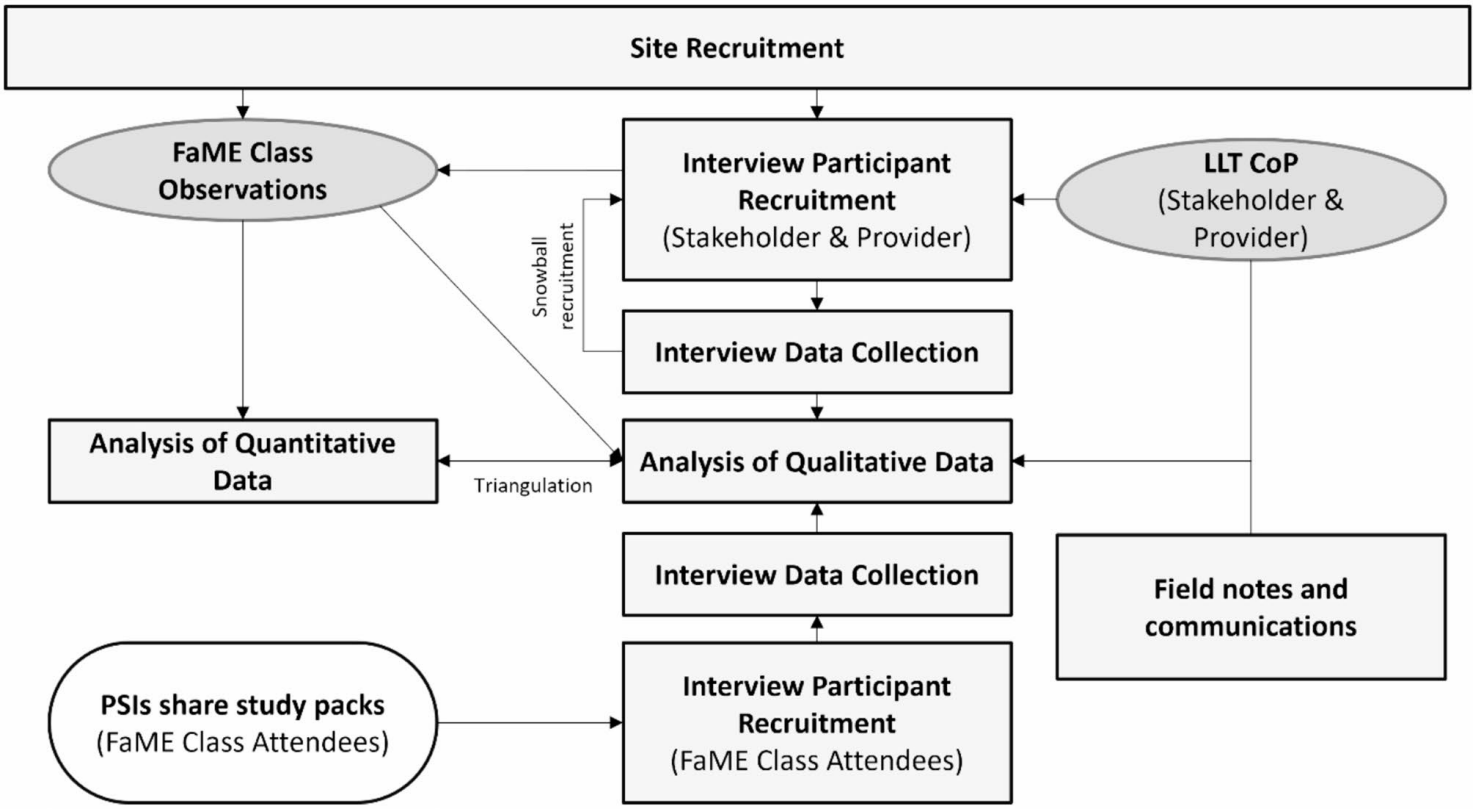
Study flowchart. Rounded white boxes indicate activity carried out by providers, grey ovals indicate routine activity carried out by Later Life Training (LLT) and grey rectangles indicate research activities. PSIs- Postural Stability Instructors. CoP- Community of Practice

To assess fidelity of FaME delivery, class observations were undertaken using a quantitative checklist. To assess influences on implementation strategy and intervention delivery fidelity, data were derived from semi-structured interviews of providers, attendees and stakeholders of FaME programmes, in vivo observations of classes, field notes, information from routine data collected in programme assessments and class registers and recordings of a national Communities of Practice (CoP) platform, hosted by Later Life Training (LLT). Implementation strategy fidelity was not evaluated against a fixed checklist but explored qualitatively, focusing on the contextual influences shaping locally adopted strategies. Intervention delivery fidelity was assessed against the National FaME Implementation Team (NFIT) list of essential components. A summary of data collection is shown in Fig. 1. LLT is a not-for-profit company, set up in 2003 that delivers the only accredited training for FaME delivery (Postural Stability Instructors (PSIs)).

### Ethics

The study received a favourable opinion from the London - City and East Research Ethics Committee REC (20/5/22, 22/PR/0634). For non-NHS sites, all sites signed data-sharing agreements between the local authorities and the research team hosted at the University of Nottingham. All interview participants provided informed consent prior to participation, and provided consent for the recording and reporting of anonymised quotes.

### Public and patient involvement and engagement (PPIE)

The study team included two PPIE representatives who contributed to study oversight and led PPIE workshops. Three online workshops were conducted to gather feedback on participant-facing documentation, findings, and dissemination. Seven PPIE members were recruited from the three study sites: five females and two males, with two each from Devon and EM, and three from GM.

### Data collection

#### Semi-structured interviews

Interviews were conducted with FaME providers (PSIs, leisure service managers, clinical leads), class attendees, and stakeholders (commissioning managers [funders of the FaME programmes], service managers, and academics [knowledge brokers]). The target sample included 15 providers, 15 class attendees, and 10 stakeholders, based on the concept of information power [14]. A mix of purposive and snowball/respondent-driven approaches were used to recruit providers and stakeholders. Class attendees were purposively sampled to reflect diverse characteristics, including locality, class provider, and gender.

Study participants received an information pack (invitation, expression of interest, consent form, and participation information sheet) in-person or via email. To support purposive sampling if required, class attendee health literacy and concern about falling were assessed at baseline using two brief screening tools. The single-item health literacy screening question (SILS) asks participants how often they need help reading health-related information [15]. The Short FES-I measures concern about falling across seven activities, with scores ranging from 7 (low concern) to 28 (high concern) [16]. Although these measures were not ultimately used to guide sampling, they are reported descriptively to characterise the participant group. Interviews were offered face-to-face or virtually (telephone/videoconferencing) with audio recording for transcription and field notes recorded live. Transcripts were not checked by study participants; however, results were discussed with PPIE representatives who had experience of FaME classes.

As part of the wider FLEXI study, semi-structured interview guides with questions and probes were developed for each participant group (see Additional file 2), influenced by the Consolidated Framework for Implementation Research [17] and expert guidance from the study team. Interviews were audio recorded, transcribed verbatim, and anonymised. Informed consent was obtained prior to the interview. Interviews were conducted by either FM, GB or JV, female researchers trained in qualitative research. The majority of participants had no established relationship with interviewers, where professional relationships existed with one interviewer (e.g. some stakeholders) one of the other interviewers conducted the data collection for objectivity.

#### FaME class observations

Two tutors, employed by LLT, conducted in vivo observations of PSIs at study sites. These were undertaken using a convenience sample of FaME classes, selected to fit the observer’s schedule and the PSIs’ willingness to participate. Observations varied in intervention timing due to differences in programme duration and time since set-up. Each PSI was observed delivering FaME once, with informed consent obtained beforehand. Feedback was given verbally and in writing and observed PSIs were able to reflect on and discuss the feedback.

Observers used a quantitative checklist based on the TiDIER framework, adapted for the FaME intervention (see Additional file 3) [18] and provided written feedback comments (qualitative data).

#### Communities of Practice (CoP)

Online national CoPs run by LLT are routinely audio recorded. These are open to providers, managers and commissioners of FaME programmes, or those looking to set up FaME programmes within the UK. These online sessions are led by the needs of the attendees, allowing sharing of best practice, problems and solutions. Recordings of these meetings were transcribed and pseudonymised with permission from attendees. Four meetings occurred during the study period and had between 8 and 28 attendees.

#### Field notes and communications

Researcher field notes, minutes/meeting notes, reports and other official communications in relation to FaME implementation were gathered. Meeting minutes, workshop outcomes and notes (including essential component analysis) and communications on programme rollout from the National FaME implementation team (N-FIT, a group of experts in FaME and falls prevention that advocate for the spread of FaME) were included.

### Data analysis

#### Qualitative data analysis

Content analysis was started while interviews and CoPs were still being undertaken. Transcripts and descriptive text from observation documents were read for familiarisation and then inductively coded before grouping into themes using NVivo 14 (QSR International). Coding was undertaken by FM, JV and AM. Coding was initially organised using the Conceptual Framework for Implementation Fidelity (CFIF), however, it was felt that the themes did not fit this framework and therefore a new conceptual map was inductively derived from the data. Regular meetings were held with study team members experienced in qualitative and implementation research (EO, JV, DAS, VG, HHH and ST) to discuss themes for reliability and consistency.

#### Quantitative data analysis

Participant demographics of gender (class attendees), location and role (stakeholders and providers) were reported as frequencies and percentages per variable. For class attendees, age and the short FES-I were described using ranges, means and standard deviations. The single item health literacy screening question (SILS) [15] was reported categorically with 4 categories of ‘*never*’, ‘*rarely*’, ‘*sometimes*’ and ‘*often*’.

Data organised using the adapted TiDIER checklist was converted into binary, where possible, of ‘included in programme’ or ‘not included in programme’. lCount data such as class size and number of weeks were described using ranges, means and standard deviations. Fidelity to the FaME programme was then assessed in line with the National FaME Implementation Team (N-FIT) list of FaME essentials.

#### Triangulation

Qualitative and quantitative data were triangulated with the aim to better understand fidelity through complementary findings. The convergent analysis was undertaken with the qualitative holding the dominant status due to the research question focussing on influences.

## Results

### Demographics

Ninety-four FaME programmes across Devon (*n* = 29), Greater Manchester (*n* = 55) and the East Midlands (*n* = 10) were included in some aspect of this study between 2021 and 23. Programmes were provided by the NHS (*n* = 25), voluntary/community sector (including charities and social enterprises) (*n* = 5) and private leisure services (*n* = 2), however, the majority were provided by a combination of providers (*n* = 51). A summary of providers and observed classes (*n* = 21) are included in Additional file 4.

#### Interview participant demographics

The target of forty interviews was achieved with 15 class attendees, 15 providers and 10 stakeholders. One interview was conducted in person at the end of the class attendee’s FaME class, all others were conducted online. Class attendees represented of health literacy levels from often, to never needing to have someone help you when you read instructions, pamphlets, or other written material from your doctor or pharmacy. They also reported varied concerns about falling from a score of 8 (low concern about falls) to 28 (high concern about falls).

Demographics of the participants are included in Table 1.

**Table 1:** Interview participant demographics

Class attendees	*n* = 15	(counts)
Location	Devon	6
	Manchester	5
	East Midlands	4
Gender	Female	10
	Male	5
Age	Range	56–84
	Mean ± SD	72.6 ± 8.21
Health Literacy (SILS)*	Never	6
	Rarely	3
	Sometimes	2
	Often	2
Concerns about falling	Short FES-I Range	8–23
	Short FES-I Mean ± SD	14.93 ± 4.48

FaME Providers		*n* = 15
Gender	Female	9
	Male	6
Role	PSI	8
	Healthcare Professional	5
	Dual PSI and Service Manager	2

Stakeholders		*n* = 10
Gender	Female	6
	Male	4
Role	Commissioning Managers (funder of FaME programmes)	3
	Academics (Knowledge broker)	2
	Service Managers	5

SILS: Single item literacy screener [15]. FES-I: Falls Efficacy Scale, International [19]. PSI: Postural Stability Instructor. *SILS asks: How often do you need to have someone help you when you read instructions, pamphlets, or other written material from your doctor or pharmacy?

### Results by theme

**Fig. 2:**
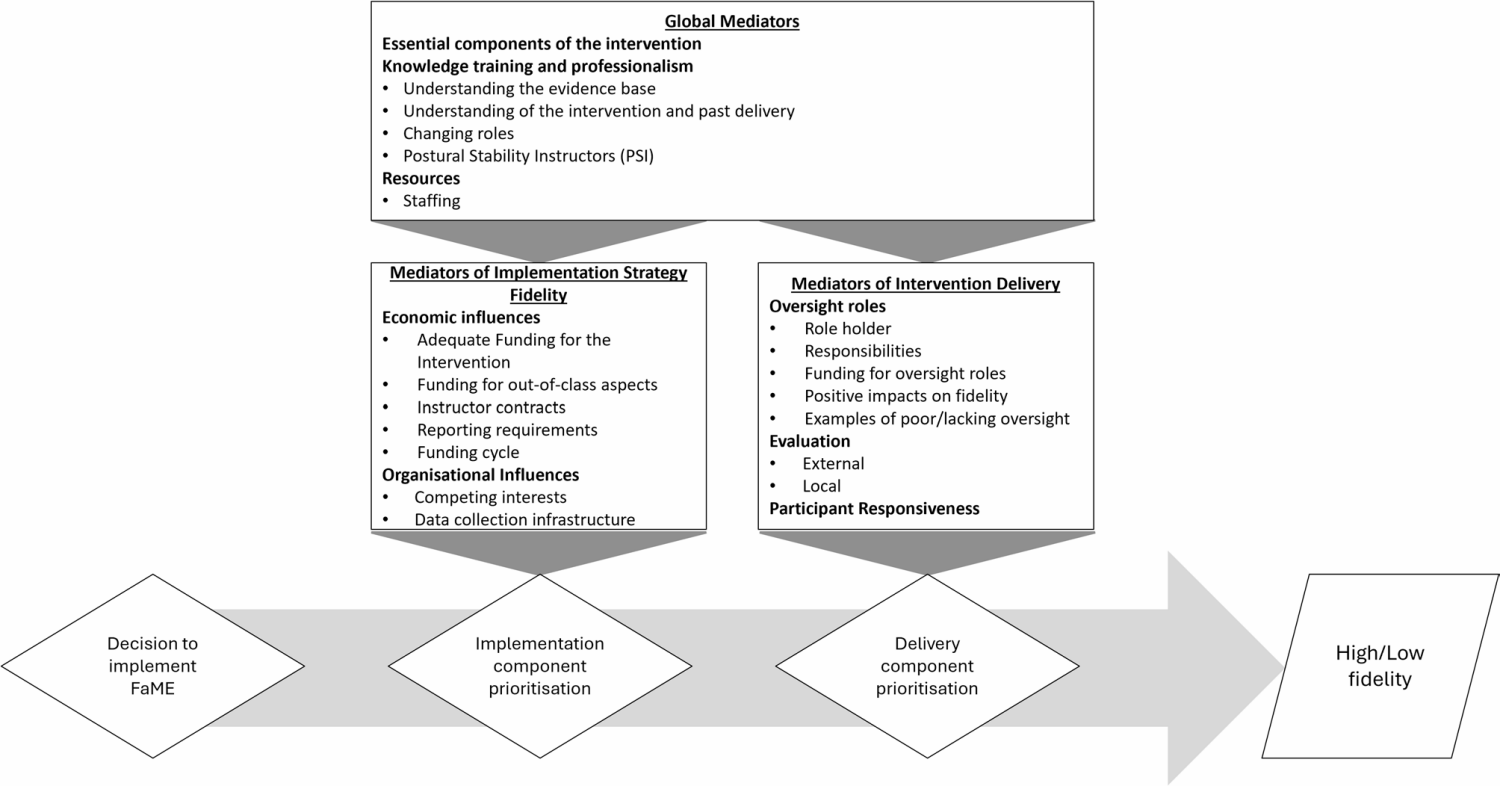
Illustrative representation of ‘Global Mediators’, ‘Mediators of Implementation’ and ‘Mediators of Delivery and Outcomes’ and their influence on prioritisation of FaME components (component prioritisation). Diamond boxes represent decisions and parallelograms represent the outcome of fidelity

The mediators of the fidelity of FaME were grouped into ‘Mediators of Implementation Fidelity’ and ‘Mediators of Delivery and Outcomes’, and mediators that impacted both were described as ‘Global mediators’. Mediators are presented in Fig. 2 with themes and indicative quotes are presented in the text and in Table 2. A mediator was defined a factor that explains a causal mechanism through which fidelity outcomes are influenced.

**Table 2:** Mediators, themes and indicative quotes

	Themes	Indicative quotes
Mediators of Implementation	**Economic Influences**	
	Adequate Funding for the Intervention	‘*It’s down as 6 sessions, which I’ve argued it before, but it used to be 12 and due to funding, … they’ve cut it. But so it’s almost it’s an impossible task, you know, really impossible’.* CoP *‘… for me is so important and that’s going to be fully funded. So they will have their 24 weeks and their home exercise, and their exercise band and their booklet and their teas and coffees and possibly their transport funded’* Provider, Interview
	Funding for out-of-class aspects	‘*we receive the money from the Clinical Commissioning Group, we weren’t allowed to charge. So what would normally happen is we would charge and then top slice of that money for tea and coffee and that would provide that or people could just pay extra. We can’t factor that in now. So it’s purely just more around advocating that that’s nice practice and that will help retain people and help with adherence*.’ Provider, Interview ‘*But the commissioning comes with the fact that …. he is paid to ensure that his instructors have CPD on a yearly basis. Appropriate CPD to FaME. So it’s not, it’s not just go along to a conference or read a book. It’s a proper, you know, it’s gotta be be*.’ CoP
	Instructor contracts	‘*If people don’t come for a few weeks, all the key things where people drop off and just needed a little bit of extra support and if they’re not costed in, if somebody’s paid on an hourly basis and that’s rush in open up the class, run it and leave, you’ve got no social time, you’ve got no, all the other bits which support the peer.*’ CoP
	Reporting requirements	‘*we are a commissioned service and from [place] public health and so, quite understandably, they want to know what we’re doing with their with their money. So there’s a delicate balance really about getting, yeah, I guess, providing what these individuals need but also demonstrating that we are, you know, we are I guess, a valued service and we’re doing what they, our Commissioner is expecting us to do’*. Provider Interview ‘*No, no, no not at all. We do … we report on … the number of participants … who attend the programme and the growth of that and that is part of our councils performance framework, but in terms of … the impact of that on the numbers and the physiological changes I suppose no we don’t have to report on that’*. Stakeholder, Interview
	Funding cycle	‘*And so we put in a business case year on year for the non-recurrent funding*.’ Provider Interview *‘And you’re right, it’s around cycles and when they happen, I mean it’s just it’s an absolute joke. It really is an absolute joke but the problem is we’re tied into government that are just in, you know, they just don’t release cash and it’s all so economically driven that it’s very, very difficult’*. Stakeholder Interview
	**Organisational influences**	
	Competing interests	‘*I mean, obviously as a charity and for [name], the tea and the coffee was always more important than anything else because that is the bit where you would be dealing with social isolation’* Provider interview
	Data collection infrastructure	‘*And I said… this is where it benefits us because we’re part of a health service … because we have to write notes anyway. So it’s not like we can get away with just scribbling on a bit of paper to say this is what they’ve done. … so we’ve already got a template set up for each group that they attend to say every person will have notes written and we’ve set up a template on the system’* Stakeholder, Interview
Mediators of Delivery and Outcome	**Oversight Roles**	
	Role holders	
	• Clinical/exercise professionals	*‘[name] is really, really experienced and she works in the hospital in the council, sorry in the city doing falls, doing physical activity therapy work etc. so she is really, really knowledgeable’* Stakeholder, Interview ‘*so they’re helping to support some of this, some of the nitty gritty of the like set up there and they’re an exercise professional by background and so that’s been really helpful to me.’* Provider, Interview
	• Non-clinical staff	*‘[name] isn’t a PSI and we had this conversation about well, how does a non-PSI support a team of PSIs? And … the concise answer is that It’s very easy to understand what they [PSIs] do. If we know with the training they’ve undertaken, what to expect within sessions, understanding fidelity and all of the reflective practice stuff that that LLT have provided, we can really ensure that actually service leads who are not PSIs can actually to a certain extent, support PSIs’* CoP
	Responsibilities	‘*So my role is supervising… Monthly supervision with the same instructors, the health trainers and we bash out problems, really. And what’s working? What isn’t? I do. We do?*’ CoP ‘*we play a kind of connecting role. So a lot of what we’re doing is brokering, sometimes funding opportunities, Sometimes connecting partners together and in the falls prevention kind of world I guess we have relationships with a number of the providers*.’ Stakeholder, Interview
	Funding for oversight roles	‘*Yeah. Like I said, the level of my involvement will depend to an extent on funding because you know that dictates … my role to an extent*.’ Stakeholder, Interview ‘*we put in a business case year on year for the non-recurrent funding and when that money comes down, we take out … a very small top slice of that for that central coordination’*. Provider Interview
	Positive impacts on fidelity	‘*And I think it was also taking on that bit of central coordination that really helped with that consistency. You know our local authority partners are fantastic, but tend to sort of go off and deliver on their own based on what their local needs are. So having that central person, … we could really make sure that we’ve got one central service specification in place*.’ Provider, Interview
	Examples of poor/lacking oversight	‘*So people were pretty much getting on with their own thing, you know, and it’s not until you go and cover a class or something that you go, oh, why’s this happening or why is that not happening and what I can do about that really… because once it’s done it’s like trying to understand it*.’ Provider, Interview ‘*But in terms of internal welfare, development, recognition, what people need, supervision, [we have] none of that. None of that is in place. You talk to about it, [they say] ‘no you don’t need that’. ‘You don’t need that rubbish’. It’s rubbish*.’ Provider Interview
	**Evaluation**	
	External	
	• CoP	‘*People respond slightly different to external people. I don’t know if that makes sense, but you know, when we running community and community of practices, so the CoP, they were obviously delivered by the researchers …. We had real buy in from a lot of the [location] partners around that*.’ Provider, Interview ‘*we’re working with later life training to create this community of practice. So, you know, I’m really keen that becomes something that instructors and potentially stakeholders can feed into and share learning and insight on so that’s one development from hoping to see*.’ Stakeholder interview
	• Observations	‘*This was a useful, but stressful experience and has already resulted in me adapting my practice, especially with regards to vocal pacing and how I sequence/teach balance activities*.’ LLT Observation, PSI Feedback ‘I did feel very deflated after my verbal feedback as it felt extremely negative**- I am sure this was unintentional but could I suggest, moving forwards, that negative feedback is balanced with some positives as I think this will be more motivating’ LLT Observation, PSI Feedback
	Local	
	• CoP	‘*so we put this WhatsApp group in place and we’ve got about 25 people on there now and it’s been fantastic really, because that was another thing I was sending emails out and a lot of these instructors are saying I’m rubbish with paperwork. I don’t look at my emails and I think you’re right. Well, what are the communication? Can we use? So we thought about our Facebook group, but not everyone was on Facebook*.’ Provider Interview
	• Observations/peer review	‘*we do quality assurance visits and pick up on stuff that’s working stuff that isn’t. So that comes into supervision. Looking at how to resolve issues.’* CoP ‘*If there was an opportunity to observe another PSI who is running a similar rolling class along these lines I would welcome this opportunity’* LLT Observation, PSI Feedback
	**Participant responsiveness**	
	Participant responsiveness	*‘We don’t do a 12 month follow up. We did do that for the first couple of years and it and we had mixed reviews…But we really struggled to get people to fill out forms or get back to us on whether they’re continuing’* Provider, Interview *‘And then let’s say you’re due to do your 12 week functional assessment. Someone doesn’t turn up, they’ll miss their assessment. So we’ve got like discrepancies in our results I suppose’.* Provider, Interview
Global Mediators	**Essential components of the intervention**	
	Essential components of the intervention	*‘I would also like Group class sizes to be in ADAPTABLE column* *Ie. - smaller necessary for lower level function and ability to support, observe, correct and provide motivational support, larger can be for higher level functioning and if have been in smaller classes before’* Stakeholder, Email correspondence *‘missing elements of FaME: No backwards Chaining or floor work’* LLT Observation, PSI Feedback
	**Knowledge**,** Training and Professionalism**	
	Understanding the evidence base	‘*And then they will start the programme and we will take them through that evidence-based programme over the course of eight sessions, we say to people it’s a minimum of eight sessions because again 8 sessions is barely skilling people up really to where we want to be. And you know obviously we know all of the evidence which is kind of looks at 50 plus hours.*’ Provider, interview
	Understanding of the intervention	‘*And erm we’re really lucky because I used to work for later life training. So I think… I suppose I get FaME, I already had a good… so I suppose I am biased from that respect and because I know how great the programme is. … so I suppose that’s helped’* Stakeholder, Interview ‘*And there’s lack of knowledge around Commissioners. Commissioners just don’t really know what their commissioning. They’ve got no real idea what their commissioning and they just think, oh, I’m commissioning falls prevention… So there’s a lot of, you know, misinformation, quite a lot of missing… miseducation as well around different types of evidence-based interventions related to falls and also an element of I just need to do that.*’ Stakeholder, Interview
	Changing roles	*‘Probably the issue that we’ve had with that group is that the chair seems to change on an annual basis, not on purpose for people moving roles and things changing. And that’s been quite difficult in terms of actually then that person coming in not knowing anything about [local programme]’.* Provider, Interview
	Postural Stability Instructors (PSI)	
	• Suitability	About a PSI with a non-exercise background: ‘[she has] *been really emotionally she… it’s was too much for her because and that’s what we’ve discovered because she’s not got those stepping stones… she didn’t have all of the knowledge to feel confident’*. Provider, Interview About PSIs: ‘*and actually [we’re pretty much all singing off the same [hymn sheet]… you know, [we] really do understand the age group we’re working with’* Provider, Interview
	• Continued professional development (CPD)	‘*but we don’t… as a charity we don’t, so I’ll be really honest with you, [name] doesn’t support professional development, doesn’t support… I mean everything we’ve asked for is a battle, everything.*’ Provider, Interview ‘*It’s quite hard to motivate people to do stuff because sometimes they don’t think there’s a need and for me, CPD keeps me fresh and interested in what I deliver because it means that I’m not always delivering the same stuff you know, I’m always looking at different ways of doing it, so it keeps it fresh as somebody who delivers physical activity’* Provider, Interview
	• Support	‘*I think is put into is going to be put into their contracts as a requirement but there’s also that checking in with each other, because working in isolation is really, really hard’* Provider, Interview ‘*And we’ve discovered even though we’ve had members of staff recently go through the PSI training, they found the session planning really, really difficult and individualising it to the participants in the class … And actually if individuals are coming out and they’re still struggling with this, and they don’t have support of their kind of you know physio service and like how do you get best practice*?’ Stakeholder, Interview
	• Mentoring	‘*And all they wanted was another PSI with them the first few times they did that [backward chaining]. And then obviously to chat to perhaps more experienced ones about certain individuals and their concerns about those individuals perhaps… And just makes you feel much more confident’*. CoP
	**Resources**	
	Resources	‘*What we have tried to do and actually we are looking to revise it this year is to make it a little bit more user friendly because I think we have got to the stage now where we’ve had some new coordinators, local coordinators come on board and they’re saying what does this mean and it’s made me rethink and think actually.*’ Provider Interview ‘*Later life provide excellent CPD that there’s absolutely no doubt about that. It’s not the only place to go but in terms of falls prevention and understanding your client group as far as I’m concerned, then my go to and you know’* Provider, Interview
	Staffing	‘*But there have been times when you’re thinking, what’s this person doing, running these classes? And I’m having to try to manage that and, you know*,’ Provider, Interview ‘*most of those classes are going to be run by newly qualified PSIs. Umm, we don’t have spare PSIs in North [Location], …, I think there are three PSIs on a current cohort and then one who’s just finishing, that are coming through*.’ Provider, Interview

### Mediators of implementation strategy fidelity

Fidelity mediators relating to implementation primarily impacted how similar the locally tailored intervention was to the trialled FaME programme through a process of prioritisation. The mediators that impacted this prioritisation were shown to be economic influences and organisational influences.

#### Economic influences

Economic influences play a key mediating role, impacted by both the national and local financial landscapes. This mediator encompasses both the funding that is available, and also the choices and behaviours that are made by commissioners and stakeholders in utilising the funding. Adequate funding for a complex intervention such as FaME is necessary. When insufficient funding is awarded, one of the aspects reported to be impacted is programme duration. Out of the 22 classes observed within this study, only 9 delivered the full 24-week programme (40.2%), with a further 9 delivering only 12 weeks, impacting on recommended dose. From qualitative data it was found that providers often had to ‘fight’ for the number of sessions they delivered (Table 2). In contrast, a pilot being run in one area was commissioned for 24 weeks including costing for equipment and home exercise booklets to specifically ensure it was delivered with fidelity. As one stakeholder highlighted:

*if … FaME was a cancer drug, no one would ever say to you “Oh, we’re only going to give you 6 weeks of this drug, even though we know you need six months to work”. No one would ever say that if it was a drug. But it seems to be acceptable to say it in relation to exercise and rehab and prevention programmes.* Stakeholder 1, Interview

Providers stated they tried to provide the ‘*best service that we can for the funding envelope*’ they received, by providing a free class for a limited number of weeks, and then offering follow-on classes that class attendees had to pay for in order to meet the minimum dose. However, even this was acknowledged as being less than ideal as ‘*not everybody then takes up the follow-on*’ jeopardising the likelihood of maintenance.

Funding, or lack of, for additional activity beyond the delivery of classes, also showed such prioritisation. Almost all classes included some behaviour change strategies (96%), but the majority (86%) relied on in-class (free) approaches such as ‘encouragement of group cohesion’ (Additional file 4). Despite this, strategies that encouraged deeper conversation, peer support and friendship such as social time before or after the class were described by providers and class attendees as key to adherence.

t*hat is what builds the rapport isn’t it of the group? Getting together and talking afterwards, yes.* Class attendee 14, Interview

Funding for additional activity also related to instructor contracts. Many PSIs are self-employed and only paid for the time it takes to deliver the class. This limits their capacity to deliver behaviour change components outside of class time such as checking-in with participants between sessions, and taking part in continuing professional development (CPD).

*So we do need to think about these things in terms of commissioning as well as real life because yeah, …, if the PSI is being paid on an hourly basis, it just doesn’t work* Stakeholder, CoP

The annual funding cycle impacted the permanence of programmes that were commissioned, with many being run as ‘pilots’. As an example, it was discussed in a stakeholder interview that a 12-month pilot programme had been commissioned to ‘*shift money from the ICB bank account’*, rather than to evaluate the programme’s suitability to the locality. Although this pilot was being delivered with high fidelity initially, it was unclear to providers if any of these aspects, or the programme as a whole was sustainable.

#### Organisational influences

It was apparent that certain providers of FaME classes had to balance the aims of FaME against the interests of the organisation. Organisations such as health providers often had aligned interests (e.g. falls prevention, hospital admission avoidance), however, some charities and leisure providers had competing or even conflicting interests. One third sector provider who originated as a ‘befriending service’ saw their primary goal as dealing with social isolation. They viewed FaME as one of their avenues to address this and emphasised the social aspects over the exercise aspects. It was also recognised that commercial providers such as leisure centres may be more motivated by ‘*bums on seats*’- fitting as many people as possible into a class - due to their own financial pressures. It was observed that this produced a deviation from the programme as tested in the trials as the motivation was not about doing what worked to prevent falls, but what brought people in and kept people in.

*whereby leisure centres, they work in a completely different… and that’s only right that if I was a leisure centre owner or whatever or director, I’d be looking for full capacity in all my classes and the maximum capacity* Stakeholder 9, Interview

Understanding of programme fidelity hinges strongly on the ability to evaluate processes and outcomes, and therefore to collect appropriate data. Despite this, the reporting requirements set out by commissioners or required by provider organisations varied greatly. Some required extensive yearly reporting, especially for programmes which had temporary funding, whereas others required little more than the number of people completing the programme.

*We report on the number of participants who attend the programme and the growth of that, and that is part of our councils performance framework but in terms of, you know, the impact of that on the numbers and the physiological changes I suppose no we don’t have to report on that.* Stakeholder 3, Interview

The data collection infrastructure of organisations also mediated fidelity and ability to evaluate the programmes. Organisations such as health care providers often had the infrastructure for and expertise in data collection, enabling assessment recording, register keeping and general demographic data collection to be easily integrated into FaME provision. However, third sector providers indicated that the need for data collection was a large burden on them. They felt it took time away from delivery and required administrative time and support that they could not provide. This led to a reduction in basic data collection, or missing data (e.g. resistance band progression) which influenced both the organisation’s ability to assess outcomes and evaluate services.

*And I said… this is where it benefits us because we’re part of a health service is because we have to write notes anyway. So it’s not like we can get away with just scribbling on a bit of paper to say this is what they’ve done … so we’ve already got a template set up* Stakeholder 4, Interview

### Mediators of intervention delivery fidelity

Observed classes were assessed against the adapted TiDIER framework, summarising the broad spectrum of delivery (Additional file 4, Table 2). Only 7/22 (31.8%) programmes observed met all of the essential FaME criteria, indicating limited fidelity (Table 3).

**Table 3:** Observed class fidelity to N-FIT list of FaME essential components

Essential Components of FaME	Count	%
PSI Trained Instructor delivering sessions/programme	21	95.5
Eligibility assessment	16	72.7
Assessment (3 time points)	12	54.5
Tailoring and progression	14	63.6
Behaviour change	12	54.5
Floor-based coping strategy	10	45.5
All exercise components*	15	68.2
Dose		
12 weeks +	22	100.0
Home exercise dose 2+ times a week^҂^	7	31.8
Meet all criteria	7	31.8

*Exercise components: Dynamic Endurance training for balance, Dynamic Balance training, Targeted Resistance training, Flexibility training and Adapted Tai Chi

҂Home exercise undertaken by class attendees was not measured; this count is assessed based on recommendations by instructors in the observed class

#### Oversight roles

Some areas employed staff with roles relating to programme oversight, and these oversight roles were influential mediators of fidelity and outcomes. Sites that had oversight roles that were well integrated, reported positive impacts on quality and fidelity, both in relation to in-class delivery, but also consistency and equity across classes. Who the oversight role holders were, and what qualifications they had, dictated the type of responsibilities they undertook. There were those who were not trained in exercise or health roles who undertook more administrative functions. These often related to ensuring training was up to date, equipment was ordered and sometimes also enabled attendee triaging to appropriate classes. Though these roles were seen as beneficial, the oversight roles that were felt to most positively affect fidelity were those undertaken by PSIs or physiotherapists. This was because they were able to offer insight into set-up, delivery and provide local quality assurance. It was clear however, that their benefit was not specifically related to their role (PSI or health care professionals) but rather to the level of their knowledge. Two providers had people in oversight roles who currently or previously worked for LLT and their extensive knowledge was seen as integral to their success.

*this is where having someone that works at Later Life Training has been really helpful … like knowing the nitty [gritty]… there’s lots of things that kind of we’re learning, but you think well, OK that’s great we’ve needed loads of support to do that*. Provider 3, Interview

The way oversight roles were integrated into organisational structures varied and was impacted by funding. Given the majority of classes had mixed provision (two or more providers delivering together, 54.3%, Additional file 4, Table 1) it is unsurprising that there were examples of collective oversight. For example, one area with several providers, including leisure and charity providers, was managed by an NHS physiotherapist employed by the commissioning organisation. Other regions utilised grouped oversight through an Active Partnership which enabled equity across classes. This role, however, was dependent upon continued funding.

*Yeah. Like I said, the level of my involvement will depend to an extent on funding because you know that dictates you know my role to an extent.* Provider 5, Interview

There were clear examples from classes where formal oversight was perceived as lacking. Participants felt that this could lead to poor practices not being identified, becoming embedded, and fidelity being lost. Coupled with the other mediators such as a lack of record keeping, deviations from the trialled intervention were often unnoticed by providers and commissioners until a chance visit or external interest such as this study.

*So people were pretty much getting on with their own thing, you know, and it’s not until you go and cover a class or something that you go, oh, why’s this happening or why is that not happening and what I can do about that really*. Provider 3, Interview

#### Quality assurance and evaluation

There was some evidence of monitoring undertaken at the local level with 59% (13/22) assessed to have internal quality assurance (QA), Additional file 4) e.g. PSI-to-PSI observations within the same organisation. However, the observation from LLT embedded in this study was the only reported method of assessment of fidelity for the remaining programmes– meaning a lack of understanding of what is actually being delivered. It should also be acknowledged that the presence of this study potentially impacted on local evaluation practices, as a stakeholder of a new programme indicated they had not set up local evaluation as they thought that the current study itself would provide fidelity evaluation and result in quality improvement.

*partly because we know that we’re going to get some quality improvement work through FLEXI study at the minute. So I haven’t worried too much about setting something up because I know that that’s… coming*. Stakeholder 4, Interview

LLT quality and fidelity evaluations though described as ‘useful’, were often cited by providers as stressful and negative. Even when positive feedback was provided, PSIs focussed on the negative/constructive elements more. However, the feedback provided was reported by some PSIs to have changed practice for the better. LLT also convened national communities of practice, which included discussions of class delivery as well as programme level or management considerations.

Local monitoring and evaluation of fidelity were more evident in areas with established oversight and relatively newer sites. For example, one area had routine observations embedded in delivery as well as a community of practice WhatsApp group. In general, there was a strong desire from providers, including PSIs, for more local fidelity evaluation and support, including the possibility to visit other classes to enable peer learning.

#### Participant responsiveness

Reasons for participant dropout were routinely collected by sites (17/22, Additional file 4) leading to a perceived threat by providers that if participants did not enjoy the sessions they would no longer come. This led to some providers reportedly omitting aspects such as assessments and home exercises due concerns it may affect attendee feedback. Both components are considered essential to enable tailoring to individual participants and to meet the minimum required dose (Table 3).

Further, fidelity to periodic assessment was strongly influenced by whether FaME class participants attended regularly. Providers found it difficult to undertake mid-programme assessments (at 6 or 12 weeks), if the participant did not attend and often the instructor was not paid to, or did not have the support to investigate absences or to carry assessments out another week.

*If you look at some services, the dropout’s 40% and a lot of that is just there’s not that wrap around to support people when they lapse or relapse to bring them back in.* Provider, COP

### Global mediators

#### Essential components of the intervention

There appeared to be a mixed understanding of the essential components of the intervention and components that were adaptable. Some aspects of FaME are considered essential for fidelity and some are considered ‘adaptable’ to meet local needs. This lack of agreement about essential/adaptable components was not only seen in providers and class attendees but also amongst those most knowledgeable about the intervention e.g. those represented by N-FIT. Captured through field notes and communications, the N-FIT team undertook a process of ‘essential components analysis’ resulting in a draft of five ‘essential’ components of FaME. There was however low agreement on these, with ongoing conversations captured by e-mail. Further, during data analysis it was realised that within the observations undertaken by LLT two different lists of essential components existed, which differed from those suggested by N-FIT (Additional file 5). Sometimes the ‘adaptable components were considered essential within checklists and other aspects of good quality teaching and delivery (quality markers) were given equal weight to the fidelity components in the checklists.

*Should we technically include venue and risk assessment in relation to the instructor/participant as ‘essential’. This should be implicit to session planning but we know it isn’t! (needs to be included in the QA)* Stakeholder, Email correspondence

*So some people think they’re doing FaME, but then when you look at it, it’s not FaME because it’s not for the right duration, or maybe there’s no quality assurance or they’re not kind of tracking people in terms of progression.* Stakeholder 6, Interview

#### Stakeholder knowledge and understanding

Commissioners in general were seen as being aware of the need for strength and balance interventions to prevent falls, however, there was a perceived lack of understanding about what this should entail.

*I suppose ensuring that we are following the guidelines that I supposed to set out from things like the NICE guidelines, really making sure that we are sort of following those as we as we need to*. Stakeholder 8 Interview

*They’ve got no real idea what they’re commissioning and they just think, oh, I’m commissioning falls prevention… So there’s a lot of, you know, misinformation, quite a lot of miseducation as well around different types of evidence-based interventions related to falls* Stakeholder 6, Interview

Sites where commissioners had good knowledge and understanding not only of the need for strength and balance, but the requirements of evidence-based FaME delivery, showed higher fidelity. Sites where commissioners reportedly lacked this, saw reports from providers of having to ‘fight’ for even basic provisions (like class size and equipment). Providers also discussed the influence of delivery organisation managers lacking an understanding of the staffing, time and equipment requirements required to deliver FaME. A solution proposed in a CoP was that people in such influential positions attend a PSI training course but this was dismissed as unrealistic due to other demands on their time.

*You know people just end up cutting corners, but you know, unless the people above you understand how much time it takes to do all these things, they don’t, they just keep giving you more to do, don’t they*? Provider 1, Interview

Linked with understanding, providers voiced frustration at the perceived constant changing roles of those in management and commissioning positions. Where effort was taken to provide education and build relationships with decision-makers, this was felt wasted when they inevitably moved on.

#### Provider knowledge, training and CPD

Even though all PSIs are trained by LLT it was noted that newer instructors were more likely to miss out on components in the classes such as backward-chaining (getting down to and up off the floor). Providers suggested that mentoring by more experienced PSIs would be beneficial to their practice and enable confidence to deliver the programme as it was trialled.

*They’ve done it in their qualification. But they hadn’t done it in real life. And all they wanted was a couple, another PSI with them the first few times they did that*. Provider, CoP

Training outside of the LLT PSI course was also reported to influence fidelity, as similar to organisational priorities, individual PSIs had their own priorities. Those with a more traditional exercise background were discussed as often working for leisure services and tended to migrate towards circuit-based classes. Those with less experience with older people prior to being a PSI appeared to focus on the seated aspects of the programme. However, those who had worked clinically, had physiotherapy backgrounds, or had other rehabilitation qualifications were believed to be able to integrate this wider knowledge to the programme’s benefit.

It was also highlighted in observations and discussed in the CoP that there is no specific requirement for continued professional development (CPD) of PSIs meaning that the availability, resources and type of CPD a PSI may engage in is organisational and individually dependent.

## Discussion

This study investigated the main influences on the implementation strategy and intervention delivery fidelity of FaME community-based falls prevention exercise classes across geographies and class delivery types. Poor fidelity to the FaME essential criteria was observed with only 32% meeting all criteria. The mediators that influenced the fidelity of this complex intervention were primarily grouped into two themes, (i) implementation mediators, (ii) delivery and outcome mediators, and a third theme which included themes that impacted at both impact and adherence called ‘global mediators’.

A key finding of this study was that there were two key phases of prioritisation that take place, affecting which components of FaME are implemented (referred to as component prioritisation). The first is at the point of implementation (and re-commissioning) and the second is during intervention delivery. The prioritisation process described in this study is similar to other fidelity frameworks proposed such as the translation ‘cloud’ described by Marielena et al. [10], that original evidence-based interventions pass through, becoming an adapted intervention. In agreement with our study, Marielena et al., found that factors such as organisational culture could influence the fidelity of implementation, acting as either barriers or enhancers [10]. However, the framework proposed by Marielena et al. [10], as well as CFIF [9] and the modified CFIF proposed by Pérez et al., [20] place the analysis of essential components only at the end of the implementation cycle, after the evaluation of outcomes and adherence. This study showed that at the point of implementation, commissioners and managers are prioritising aspects of the intervention, with these decisions mediated by knowledge (or lack) of the intervention, economic influences and organisational priorities. Adaptation of evidence-based interventions is key to improving their fit in a new context, however, essential components should not be adapted as without these, intervention effectiveness cannot be guaranteed [21]. A recent UK wide survey of FaME delivery (247 PSIs) most were running 6–12 week programmes with classes as large as 30 people due to funding constraints [22]. Our study highlighted that information on and understanding of essential components of FaME needs to be available at the point of implementation, in order to support commissioners and managers to maintain implementation fidelity, as well as ensure adequate funding is secured for the delivery of the intervention.

The general lack of clarity on the essential components of the FaME intervention influenced both implementation and providers’ ability to assess adherence. The clarity of essential components has been widely cited as impacting on delivery [23, 24], yet programmes are still being developed and translated into practice without essential components agreed. The development of implementation toolkits has helped bridge the translation of interventions into practice [25]. FaME has such an Implementation Toolkit [26] developed from the PhISICAL study [11], which includes the importance of essential components e.g. 24 week delivery and home exercise to meet dose. However, where provision existed prior to the existence of the toolkit combined with poor or lacking oversight (Table 2), migration of delivery away from the evidence base was evident.

Oversight roles also mediated delivery adherence at a programme and class level. Where those in oversight positions had funding, capacity and expertise related to the intervention (e.g. PSI or physiotherapist), fidelity was perceived as higher. Similar findings have been shown in a recent study about PSIs, where it was reported by one participant that they did not include the adapted Tai-Chi aspect of FaME in their classes as their ‘*manager doesn’t like to … she has religious reasons why she feels it’s wrong’* [22]. Effective local governance was especially key when delivery was split between providers. During this study, a new set of providers started delivering FaME in one area and whilst they had a mix of leisure and charity providers, they had oversight from an NHS physiotherapist. This role acted as a liaison between sites and ran a local CoP enhancing standardisation and quality. Integration across providers is not easy, especially with competing interests, differing communication styles and mistrust between professionals [27]. The clear reliance on voluntary and community sector care, specifically in the case of older adults is likely to continue [28], meaning integration of delivery and effective local governance is key to enabling effective local programmes of consistent fidelity.

Local governance and a clear understanding of essential components was also linked to the ability to evaluate programmes. Within this study there were varying models of local monitoring and evaluation, frequently influenced by organisational priorities or participant responsiveness. Where oversight role holders were engaged and knowledgeable about the intervention, there was evidence of good awareness of delivery fidelity. Without such insight, the focus in some programmes were motivated by what kept participants attending, over and above what has been evidenced to produce the targeted health outcomes. There was a strong desire from providers, including PSIs, for more local evaluation and support, however, clarity on what should evaluated is needed. In recognition of this, a study on the implementation of a Fitness and Mobility Exercise Program for stroke [29] utilised an adaptation of the TiDIER checklist as a training and audit tool that clarified ‘key active ingredients’ [30]. The study followed the programme for 1 year, which included 3 iterations of the 12 week programme, and not only reported high fidelity but increasing fidelity [30]. Although an adapted TiDIER checklist was utilised within our study as part of the observations, its use as a framework for the embedding of training and audit of essential components could be explored in future work, including its use in PSI training and Toolkit resources.

This study was limited somewhat in its ability to understand delivery fidelity other than through routinely-delivered observations and self-report in interviews. The finding of low levels of local fidelity evaluation meant that sites and providers had limited knowledge and evidence of their own programme adherence. As well as a likely response bias, it is plausible that there may have been an observer effect, PSIs changing their normal practice due to the knowledge of being observed, meaning it is likely the observed fidelity was higher than routine classes. The embedding of routine and consistent local fidelity evaluations would therefore not only benefit local oversight and commissioning decisions but would also allow key research insights into routine fidelity practices. This limitation was reduced somewhat by the triangulation of data from a range of sources including routine communities of practice, interviews and communications.

## Conclusion

We aimed to investigate the key factors influencing the fidelity of FaME programmes using a mixed-methods approach. Through the development of a new framework for implementation fidelity, we identified mediators as pivotal both in the implementation and delivery of complex interventions, as well as in their broader contextual role. Our findings reveal that FaME classes are not always delivered in accordance with the evidence base due to prioritisation decisions influenced by funding, organisational priorities and knowledge among others.

To ensure programmes are implemented and maintained with fidelity and evaluated effectively, it is critical to communicate the essential components of the programme at all levels. A standardised tool should be developed to measure the delivery of these components, with monitoring requirements embedded into local service specifications. This approach will support the alignment of local delivery with evidence-based practice, safeguarding the integrity and impact of FaME interventions.

## Supplementary Information

Below is the link to the electronic supplementary material.

Supplementary Material 1

Supplementary Material 2

Supplementary Material 3

Supplementary Material 4

Supplementary Material 5

## Data Availability

The datasets generated and/or analysed during the current study are not publicly available due to participants not providing expressed permission for their data to be shared in this way, however, are available from the corresponding author upon reasonable request.

## Abbreviations

FaME: Falls Management Exercise programme
PSI: Postural Stability Instructor
CFIF: Conceptual Framework for Implementation Fidelity
UK: United Kingdom
GM: Greater Manchester
EM: East Midlands
CoP: Community of Practice
LLT: Later Life Training
PPIE: Patient and Public Involvement and Engagement
SILS: Single item health literacy screening
FES: I-Falls efficacy scale-international
TiDIER: Template for Intervention Description and Replication
N: FIT-National FaME Implementation Team
CPD: Continued Professional Development
