# Supplementary material for: Mediators implementation and delivery: the falls management exercise programme (FaME)
Source: BMC Health Serv Res. 2025 Oct 22;25:1396. doi: 10.1186/s12913-025-13550-7 (PMC12542040; doi:10.1186/s12913-025-13550-7)
Supplement: Supplementary file 2 — Supplementary Material 2 [file 12913_2025_13550_MOESM2_ESM.docx]

Supplementary Material 2- Interview Guides

1. **FLEXI Providers semi-structured interview topic guide**

**You, and your role**

- Tell me about your organisation and or role in it, background in falls prevention or related area? (prompt: other experience, years in position, passions)
- **For PSIs** tell me how long you have been a PSI and about the organisation/s you have provided FaME instruction for and your role within it (prompt: providing for public or voluntary sector; experience with different providers)

**Implementation and adoption**

- Tell me about your local falls exercise programme/service (prompt: is it based on a particular evidence based programme; when was it set up; are there core components that you include or not; how decided, COVID)
- Can you tell me about the pathways into falls exercise provision in your area? (prompt: criteria, referrers, signposting, follow on classes, other opportunities, key links)
- Talk me through any assessments you carry out as part of the programme? (prompt: timings; challenges)
- Can you tell me about your approach to FaME and your PSI classes (prompt: what a typical class is like/entails, progression, hybrid?)
- How do you support people during and outside your classes (prompt: any strategies; behaviour change; homework; written exercises; peer support; phone calls, diary, social)
- How do you monitor quality of the programmes, and your own fidelity & continuous improvement (prompt: observations, discussion groups, feedback, reviews, CoP)

**Does the programme work**

- How effective do you feel your programme/service is? (Prompt: how do you evaluate, service user views)?
- What has worked well in delivering FaME? Have you adapted it at all (prompt: what, why, how, impact)
- What have been the challenges so far? Have you found ways to overcome them; Do you see different challenges moving forwards?

**For Service Providers only:**

- Can you describe procurement/tendering/commissioning process (prompt: Why did you bid/competitors, reasons for success).
- What did and didn’t work implementing/delivering FaME in your area? (prompt: observations, who and why were influential, any modifications, did you need to negotiate changes)
- What are your plans to continue FaME or a version of it in the future? Why? How will you do this? (prompt: funding, models, etc)

**Closing remarks**

- Anything else you would like to share with me
- Are there key people you think we should approach to interview

1. **FLEXI Stakeholders’ semi-structured interview topic guide**

**You, and your role**

- Tell me about your organisation and your role within it (prompt: other experience, years in position, passions)
- How does your role relate to others in falls prevention in your area, region, nationally? (prompts: are they influencer, how do they relate to CCGs, councils, charities, government, private providers)

**Implementation and adoption**

- Tell me about what is available in your area/service in terms of falls prevention exercise programmes (prompts: is it based on a particular evidence based programme; how was this decided)?
- How did this particular service come about? (prompt: has it always been the same; how has it evolved/changed over time; who has been involved; when did it start; why did it start)
- What sorts of things have contributed to the service development and how has it helped? (prompt: people; organisations; services; policy; guidelines; relationships; toolkit)
- What sort of things have hindered progress? (Prompt: things that didn’t happen or go as planned; unforeseen circumstances)? What impact did this have?
- How do you think service users view the programmes and the ways they are run? Why?
- Is there anything about your service you would change? If yes: can you please describe in what circumstances you might do this? And why?
- What could others learn from your service and how it has developed in order to develop their own service

**Does the programme work?**

- How effective do you feel your programme/service is? (Prompt: how do you evaluate)?
- Is there anything you would do differently to enable more/different people to take part (prompt: health inequalities)
- What is stopping you offering the programme/service to all those at risk of falling in your area? What could be done to overcome these issues?

**Closing remarks**

- Anything else you would like to share with me
- Are there key people you think we should approach to interview?

1. **FLEXI Class Attendees semi-structured interview topic guide**

- Can you tell me about how you came to start at these classes (what happened, how did you hear about it, referral process etc)
- Can you tell me about the classes, what happens, what you like and don’t like?
- Was the class what you expected? What did you want from it/did you get what you wanted?
- Has attending the classes had an impact on other aspects of your life? (activity levels, confidence v. fear)
- Were there any barriers to you attending the classes? Why? How were they overcome?
- Have your classes or your feelings about attending the classes been affected by COVID?
- Would you recommend your class to anyone else? Why/why not?
- Are home-exercises part of the exercise programme? (prompt, what do you think about them, do you think they could be useful, what support might you need to do them?)
- Do you think there is anyone the programme wouldn’t be suitable for? Why?
- What do you think could be better about the programme? Why? How?

**Closing remarks**

- Anything else you would like to share with me?
- Are there key people you think we should approach to interview?

**Signposting:**

General support and advice

- Age UK Advice Line on 0800 678 1602
- Silver Line 0800 4 70 80 90
- Independent Age 0808 503 7945
- Citizens Advice 0800 144 8848

Mental Health

- Samaritans 116 123

Domestic Abuse

- Refuge 0808 2000 247
