# Supplementary material for: Mediators implementation and delivery: the falls management exercise programme (FaME)
Source: BMC Health Serv Res. 2025 Oct 22;25:1396. doi: 10.1186/s12913-025-13550-7 (PMC12542040; doi:10.1186/s12913-025-13550-7)
Supplement: Supplementary file 3 — Supplementary Material 3 [file 12913_2025_13550_MOESM3_ESM.docx]

**Wider scale-up of the Falls Management Exercise (FaME) programme**

**FLEXI implementation and cost analysis study**

**TIDIER Checklist**

**Aim: to document key elements of the FaME programme delivered**

| **Item** |  | **Item** | **Any online information / links / URLs?** |
| --- | --- | --- | --- |
|  | **Programme / Session**  **Name** | Eg. Staying Steady |  |
| **2.** | **Aim and / or goals of the Programme / Session** | Eg. to reduce falls, to improve confidence, to improve steadiness and strength  Note both public facing description and that given to partners/HPs |  |
| **3.** | **Materials and programme components** | Sessions based on FaME:  🞎 Yes  🞎 Yes but missing elements of FaME (which ones?________________________________)  🞎 No  Cost to attend:  🞎 Yes £______________________  🞎 No, free  □ Free for _________ weeks/sessions. Cost £_____________ after  Transport provided:  🞎 Yes  🞎 No  Resources given to participants:  🞎 Home exercise sheets/booklets  🞎 Home exercise monitoring sheets  🞎 Other information booklets on health  🞎 Online follow me videos  🞎 Online home exercise illustrations  🞎 Home exercise DVD  □ Exercise equipment (resistance bands/weights)  🞎 Other: _________________________  Behaviour change components present:  🞎 Self-monitoring (home exercise diary/functional tests to check themselves etc)  🞎 Social time before or after  🞎 Educational sessions in addition (on other falls topics or physical activity)  🞎 Improving physical literacy about falls and exercise (within session)  🞎 Encouraging group cohesion  □ Other: _______________________________ |  |
| **4.** | **Procedures** | Entry to the programme:  🞎 Referral from falls service NHS  🞎 Referral from elsewhere: Where:_______________________  🞎 Self-refer / drop-in  Pre-exercise assessment for tailoring by PSI:  🞎 pre-exercise assessment identifying suitability  □ pre-exercise assessment to identify baseline exercises and tailoring  □ pre-exercise assessment of motivation and behaviour change support requirements  🞎 No assessments  🞎 No but an assessment is done by someone else: Who:_______________________  Service outcome measures:  🞎 Attendance – class / session register  🞎 Functional Grid  🞎 Functional Tests: ____________________________  🞎 Questionnaires:______________________________  🞎 Other:________________________________________ |  |
| **5.** | **Session providers** | Alongside PSI:  🞎 Other trained exercise instructor  🞎 Volunteer  🞎 Other:______________________ |  |
| **6.** | **Modes of delivery** | Delivered face to face or virtual:  🞎 Face to face only  🞎 Virtual only (live or recorded?) State which:____________________  🞎 Both offered  Delivered in groups or on a one to one basis:  🞎 Group sessions  🞎 One to one  If Group, usual participant to instructor ratio: _____ _: 1  If Group, participant flow:  🞎 Cohort  🞎 Rolling Group  If Group, format type:  🞎 Chairs in lines  🞎 Chairs in circles  🞎 Circuit formats  🞎 Mixed: __________________ |  |
| **7.** | **Location** | 🞎 Community Centre  🞎 Leisure Centre  🞎 Therapy Setting  🞎 Other:___________________ |  |
| **8.** | **Intervention schedule, frequency and dose** | Duration of programme (for participant, whether cohort or rolling programme): __________________________  PSI led:  🞎 Once per week  🞎 Twice a week  🞎 Three times a week  Home Exercise:  🞎 Not encouraged  🞎 Twice a week  🞎 Three times a week  🞎 Home exercise integrated into daily activities | _ |
| **9.** | **Individual tailoring** | 🞎 Evidence seen in session  🞎 Evidence seen in home exercise programme  🞎 Evidence seen in virtual delivery modes | _ |
| **10.** | **Modifications** | **FaME Adaptables:**  🞎 Group class size (very large as lower risk participants)  🞎 Rolling Groups (as opposed to cohorts)  🞎 Floorwork included  🞎 Tai Chi included  🞎 Equipment use:_____________________________  🞎 Step down programme to transition to  🞎 Ability to refer back to physiotherapy  □ Layouts  □ Format of circuit design | - |
| **11.** | **Intervention acceptability and fidelity (planned)** | N/A | - |
| **12.** | **Intervention acceptability and fidelity (actual)** | **Fidelity:**  **FaME Essentials:**  🞎 PSI Trained Instructor delivering sessions/programme  🞎 Assessment of ability and needs of participant prior to programme starting  🞎 Ongoing assessment (or at end at least) of participant  🞎 Getting down to and up from floor included  🞎 Behaviour change to support motivation and adherence  🞎 Internal QI and measurement strategy  🞎 LLT QI feedback form completed  Acceptability  🞎 Participant feedback on acceptability gathered by programme  🞎 Reasons for drop out considered  🞎 Other |  |
