# Supplementary material for: Mediators implementation and delivery: the falls management exercise programme (FaME)
Source: BMC Health Serv Res. 2025 Oct 22;25:1396. doi: 10.1186/s12913-025-13550-7 (PMC12542040; doi:10.1186/s12913-025-13550-7)
Supplement: Supplementary file 4 — Supplementary Material 4 [file 12913_2025_13550_MOESM4_ESM.docx]

**Supplementary Material 4**:

Table 1. Number of classes per region and provider

| **Total Classes** | **94** | **Classes N (percentage)** |
| --- | --- | --- |
| **Region** | Greater Manchester | 55 (58.5) |
|  | Devon | 29 (30.9) |
|  | East Midlands | 10 (10.6) |
| **Provider** | **Solo Provider** | **43 (45.6)** |
|  | NHS | 25 (26.6) |
|  | Private Leisure Service | 3 (3.2) |
|  | Third Sector | 5 (5.3) |
|  | Local Government Leisure Service | 10 (10.6) |
|  | **Shared Provision** | **51 (54.3)** |
|  | NHS & Private Leisure Service | 2 (2.1) |
|  | NHS & Third Sector | 17 (18.1) |
|  | NHS & Local Government Leisure Service | 3 (3.2) |
|  | Private Leisure Service & Third Sector | 17 (18.1) |
|  | NHS, Private Leisure Service & Third Sector | 12 (12.8) |

Table 2. Counts of observations based on the adapted TiDIER framework.

|  | **Total times observed** | **Percentage (%)** | **Mean ± Standard Deviation** |
| --- | --- | --- | --- |
| **Locations** |  |  |  |
| Devon Observations | 5 | 22.7 |  |
| Manchester Observations | 9 | 40.9 |  |
| East Midlands Observations | 8 | 36.4 |  |
| **Sessions based on FaME** | 15 | 68.2 |  |
| Yes, but missing components | 7 | 31.8 |  |
| **Cost to attend** | 1 | 4.6 |  |
| *free then cost to attend* | *1* | 4.6 |  |
| Cost to participants |  |  | 7 ± 1.41 |
| **Transport provided** | 0 | 0 |  |
| **Resources given** |  |  |  |
| Home exercise sheets/booklets | 14 | 63.6 |  |
| Home exercise monitoring sheets | 0 | 0 |  |
| Other information booklets on health | 0 | 0 |  |
| Online follow me videos | 0 | 0 |  |
| Online home exercise illustrations | 0 | 0 |  |
| Home exercise DVD | 0 | 0 |  |
| Exercise equipment (resistance bands/weights) | 7 | 31.8 |  |
| **Behaviour change components present:** | **21** | 95.5 |  |
| Self-monitoring (home exercise diary/functional tests to check themselves etc) | 11 | 50 |  |
| Social time before or after | 1 | 4.6 |  |
| Educational sessions in addition (on other falls topics or physical activity) | 1 | 4.6 |  |
| Improving physical literacy about falls and exercise (within session) | 19 | 86.4 |  |
| Encouraging group cohesion | 19 | 86.4 |  |
| **Entry to the programme** |  | 0 |  |
| Referral from falls service NHS | 21 | 95.5 |  |
| Referral from elsewhere | 14 | 63.7 |  |
| Where |  |  |  |
| Self-refer / drop-in | 13 | 59.1 |  |
| **Pre-exercise assessment for tailoring by PSI:** |  |  |  |
| pre-exercise assessment identifying suitability | 16 | 72.7 |  |
| pre-exercise assessment to identify baseline exercises and tailoring | 13 | 59.1 |  |
| pre-exercise assessment of motivation and behaviour change support requirements | 4 | 18.2 |  |
| No assessments | 1 | 4.6 |  |
| No but an assessment is done by someone else* | 5 | 22.76 |  |
| *Who |  | Percentage of 5 |  |
| *Physio* | 2 | 40 |  |
| *Referring org* | 1 | 20 |  |
| **Service outcome measures:** |  |  |  |
| Attendance – class / session register Y/N | 22 | 100 |  |
| Functional Grid | 16 | 72.7 |  |
| **Functional Tests Y/N** | 5 | 22.7 |  |
| Type of tests done |  | Percentage of 5 |  |
| *30sec sit to stand* | 2 | 40 |  |
| *Timed up and go* | *5* | 100 |  |
| **Questionnaires Y/N** | 17 | 77.3 |  |
| Questionnaires used |  | Percentage of 17 |  |
| *FES-I* | *1* | 5.9 |  |
| *BERG* | *2* | 11.8 |  |
| *Fear of falling* | *2* | 11.8 |  |
| *CONFBAL* | *8* | 47.1 |  |
| *FRAT* | *11* | 64.7 |  |
| **Staff alongside PSI:** |  |  |  |
| Other trained exercise instructor Y/N | 1 | 4.6 |  |
| Volunteer | 2 | 9.1 |  |
| Other | 7 | 31.8 |  |
| **Delivered face to face or virtual:** |  |  |  |
| Face to face only | 20 | 90.9 |  |
| Virtual only (live or recorded?) | 0 | 0.00 |  |
| Both f2f and virtual offered | 1 | 4.6 |  |
| Live | 1 | 100 |  |
| **Delivery basis** |  |  |  |
| Group* | 22 | 100 |  |
| 1 to 1 | 0 | 0 |  |
| * usual participant to instructor ratio: _____  _: 1 |  |  | 9.92 ± 4.52 |
| If Group, participant flow: ROLLING | 13 | 59.1 |  |
| If Group, participant flow: COHORT | 9 | 40.9 |  |
| **Location** |  |  |  |
| Community Centre | 15 | 68.2 |  |
| Leisure Centre | 6 | 27.3 |  |
| Therapy Setting | 0 | 0 |  |
| **Duration of programme (for participant, whether cohort or rolling programme)** | | | |
| Length average weeks |  |  | 15.27 ± 5.61 |
| 12 weeks | 9 | 40.2 |  |
| 24 weeks | 9 | 40.2 |  |
| Open ended | 4 | 18.9 |  |
| **PSI led** |  |  |  |
| Once per week | 22 | 100 |  |
| Twice a week | 0 | 0 |  |
| Three times a week | 0 | 0 |  |
| **Home Exercise** |  |  |  |
| Not encouraged | 6 | 27.3 |  |
| Twice a week | 1 | 4.6 |  |
| Three times a week | 6 | 27.3 |  |
| No mention of dose | 10 | 45.5 |  |
| Home exercise integrated into daily activities | 13 | 59.1 |  |
| **Individual Tailoring** |  |  |  |
| Evidence seen in session | 14 | 63.6 |  |
| Evidence seen in home exercise programme | 3 | 13.6 |  |
| Evidence seen in virtual delivery modes | 0 | 0 |  |
| **Adaptables** |  |  |  |
| Group class size (very large as lower risk participants) | 6 | 27.3 |  |
| Rolling Groups (as opposed to cohorts) | 12 | 54.6 |  |
| Floorwork included | 10 | 45.5 |  |
| Tai Chi included | 15 | 68.2 |  |
| Equipment use | 22 | 100 |  |
| **Equipment used** |  |  |  |
| Bands | 20 | 90.9 |  |
| Mats | 13 | 59.1 |  |
| Chairs | 20 | 90.9 |  |
| Step down programme to transition to | 2 | 9.1 |  |
| Ability to refer back to physiotherapy | 4 | 18.2 |  |
| **Essentials** |  |  |  |
| PSI Trained Instructor delivering sessions/programme | 21 | 95.5 |  |
| Assessment of ability and needs of participant prior to programme starting | 15 | 68.2 |  |
| Ongoing assessment (or at end at least) of participant | 12 | 54.6 |  |
| Getting down to and up from floor included | 10 | 45.5 |  |
| Behaviour change to support motivation and adherence | 5 | 22.7 |  |
| Internal QI and measurement strategy | 13 | 59.1 |  |
| total/6 |  |  | 3.45 ± 2.11 |
| **Acceptability** |  |  |  |
| Participant feedback on acceptability gathered | 9 | 40.9 |  |
| Reasons for drop out considered | 17 | 77.3 |  |
