# Supplementary material for: Mediators implementation and delivery: the falls management exercise programme (FaME)
Source: BMC Health Serv Res. 2025 Oct 22;25:1396. doi: 10.1186/s12913-025-13550-7 (PMC12542040; doi:10.1186/s12913-025-13550-7)
Supplement: Supplementary file 5 — Supplementary Material 5 [file 12913_2025_13550_MOESM5_ESM.docx]

Supplementary Material 5- Conflicting essential fidelity components grouped by theme. Items included from assessment documents and programme descriptions that were listed as ‘essential’. Empty boxes indicate this aspect is not included as an ‘essential’ item, however may have still been included in an assessment or description. Clarifying points are included in brackets and italics.

| **Topic** | **Later Life Training observation  (fidelity questions)** | **Adapted TiDIER FaME Essentials (SM3)** | **N-FIT FaME Essentials** |
| --- | --- | --- | --- |
| **Instructor training** | *(Instructor would not be assessed if not PSI trained)* | PSI Trained Instructor delivering sessions/programme | PSI trained lead* instructor- delivering FaME program (1999 paper on FaME essentials) |
| **Assessment** | *(assessment classified as quality rather than fidelity)* | Assessment of ability and needs of participant prior to programme starting (pre-exercise assessment) | Assessment of the ability and needs of participants at the beginning and throughout the programme |
|  |  | Ongoing assessment of ability, need and progression (or at end at least) of participant |  |
| **Tailoring** | Selected safe and effective exercises appropriate to all FaME components and in relation to the group/individuals. | *(Tailoring included in TiDIER questions, however not classified as ‘essential’)* |  |
|  | Adapted exercises to meet the needs of the participants with postural stability challenges |  |  |
|  | Selected safe and effective exercises appropriate to the stage in the intervention |  |  |
|  | Offered alternatives to allow for different levels of ability/ tailored exercises to individuals |  |  |
| **Instructor skills** | Provided specific and relevant teaching points to enhance technique, effectiveness and postural stability |  |  |
|  | Provided safe transitions between exercises and session components |  |  |
| **Behaviour change** | Engaged participants in order to motivate and promote confidence throughout all components | Behaviour change to support motivation and adherence | Behaviours Change to support motivation and adherence to the programme and self-literacy to continue after programme cessation (individual and local setting) |
| **Quality assurance and improvement** | *(((observation completed as part of external quality assurance)* | Internal QI and measurement strategy | Internal quality assurance & measurement strategy |
| **Dose*** |  |  | Dose- 3x per week up to 2hrs total |
| **Home Exercise*** | Provide Home exercise Programmes (HEP) and Include into discussion and teaching in a meaningful educational way |  |  |
| **Backward chaining down to and up from floor included** | Floorwork (if applicable) | Getting down to and up from the floor included |  |

**Dose and home exercise closely related. In most 1x week classes, home exercise required to meet dose.*
